# Power laws in the Roman Empire: a survival analysis

**DOI:** 10.1098/rsos.210850

**Published:** 2021-07-28

**Authors:** P. L. Ramos, L. F. Costa, F. Louzada, F. A. Rodrigues

**Affiliations:** ^1^ Institute of Mathematics and Computer Science, University of São Paulo, São Carlos, Brazil; ^2^ São Carlos Institute of Physics, University of São Paulo, São Carlos, Brazil

**Keywords:** power-law, Roman Empire, survival analysis

## Abstract

The Roman Empire shaped western civilization, and many Roman principles are embodied in modern institutions. Although its political institutions proved both resilient and adaptable, allowing it to incorporate diverse populations, the Empire suffered from many conflicts. Indeed, most emperors died violently, from assassination, suicide or in battle. These conflicts produced patterns in the length of time that can be identified by statistical analysis. In this paper, we study the underlying patterns associated with the reign of the Roman emperors by using statistical tools of survival data analysis. We consider all the 175 Roman emperors and propose a new power-law model with change points to predict the time-to-violent-death of the Roman emperors. This model encompasses data in the presence of censoring and long-term survivors, providing more accurate predictions than previous models. Our results show that power-law distributions can also occur in survival data, as verified in other data types from natural and artificial systems, reinforcing the ubiquity of power-law distributions. The generality of our approach paves the way to further related investigations not only in other ancient civilizations but also in applications in engineering and medicine.

## 1. Introduction

As complementation to continuing studies by historians, anthropologists and social scientists, ancient civilizations can be analysed quantitatively, considering mathematical and computational modelling [1,2]. Ancient civilizations are examples of a complex system composed of agents that interact, collaborate, and compete for power and resources. One example of such a system is the Roman Empire, which started with Augustus (d. 14 CE) and ended with Romulus Augustulus, with the Germanic invasion from the North in 476. After this, the eastern Roman Empire, with Constantinople as its capital, continued to exist until 1453 [3]. The Roman Empire influenced western civilization and contributed to many aspects of our culture [4]. Although the first two centuries of the Empire were characterized by stability and prosperity, known as the Pax Romana, the Roman Empire underwent several crises, including many violent deaths of Roman emperors. A recent study suggested that among the 69 rulers, 43 emperors suffered a violent death [5], including homicide, suicide or death in combats with a foreign enemy of Rome. These conflicts produced patterns in the length of time that can be identified by statistical analysis, as we show in this paper.

We propose a power-law model to describe the time-to-violent-death, which measures the time before a Roman emperor suffers a violent death since the beginning of his reign. This variable is interesting from the historical point of view as it can suggest some patterns that governed the dynamics of the Roman Empire. For example, if deaths occur independently, without any defined rule, we should observe an exponential distribution of this survival time, where the hazard function is constant. Otherwise, some other distribution should be more suitable to model the time-to-violent-death. Recently, Saleh [5] analysed the time-to-violent-death of the 69 emperors of the unified Roman Empire, from Augustus (d. 14 CE) to Theodosius (d. 395 CE). The author considered statistical tools of survival data analysis to model their time-to-violent-death and verified that a mixture of two Weibull distributions captures this variable well. However, as we show here, that model does not seem to be easily extended to the long-term perspective observed for some Roman emperors who did not face a violent death. Moreover, the dataset analysed covers only part of the Roman emperors, leaving out, for instance, the Byzantine Empire. To address these limitations, we show that a power-law distribution with change points can better model the time-to-violent-deaths. This power-law behaviour in time distribution has been observed in many complex systems, including bacterial persistence [6], earthquakes [7–9], in solar radiophysics [10], stock price fluctuations [11] and tree-limb branching [12]. The cause of this distribution is related to the presence of extreme events, such as large earthquakes. In the case of the Roman Empire, we observe that an extreme event is an emperor surviving for many years since the beginning of his reign. Our model shows that most of Rome’s emperors suffered violent deaths during the initial years of their reign.

We study the distribution of time-to-violent-death based on survival analysis, which refers to a set of statistical approaches used to investigate the time it takes for an event of interest to occur [13]. The term survival analysis originates from clinical research, where predicting the time to death, i.e. survival, is often the primary goal. The maximum-likelihood estimator (MLE) is used to estimate the best distribution for all the considered emperors, considering data in the presence of censoring and long-term survivors. Indeed, the inclusion of long-term survival enables a more accurate prediction regarding the emperors who did not have a violent death than in the study by Saleh [5]. By considering the dataset by Saleh [5], we also verify that the risk after assuming the throne is very high for a Roman emperor, but this risk systematically decreases until 13 years of rule and rapidly increases after this change point. This change point indicates a change in the Roman Empire’s tendency and dynamics after a given time of reign. Further, we also consider the Byzantine Empire’s time-to-violent-death that, combined with the initial data, comprehends 175 emperors. In this case, we observe a more complex behaviour with additional change points and, therefore, we generalize the power-law model to include multiple change points.

Our model is mainly general and can be used in any system in which the reliability function follows a power law, and we have censored data. Indeed, our work opens the possibility of studying empires and dynasties by using statistical analysis tools, contributing to the understanding of social and historical sciences more quantitatively.

This paper is organized as follows. Initially, we consider the data by Saleh [5] and show that the power-law model is more suitable to model the distribution of time-to-violent-death than previous models. We identify a change point that occurs at 13 years of reign. Next, we consider a dataset of 175 Roman emperors, including also the Byzantine Empire. In this case, we introduce a generalized power-law distribution with *k* − 1 change points and show that this distribution accurately describes the data observed. Finally, we analyse some possible causes of the short lives of Roman emperors and verify that the birthright is a statistically significant variable, whereas other features, such as the birth province and dynasty, do not influence the time-to-violent-death. These findings suggest that emperors who inherited their reign tend to have a more peaceful reign and increased probability of natural death. At the end of the paper, we present the mathematical formulation and simulation study related to our statistical model.

## 2. Survival analysis

The power-law distribution can be represented by its survival function (also refereed by complementary cumulative density function) given by

$$S(t)={\left(\frac{t}{t_{\min}}\right)}^{1-\alpha },$$

where *α* > 1 and *t*_min_ > 0 is the minimum value at which power-law behaviour holds. The inferential methods to estimate *α* have been discussed by many authors [14–16]. The most common approach to this end is the MLE, which in the continuous case has a simple closed-form expression as well as desired properties, such as consistency and asymptotically efficiency.

**Figure 1.**
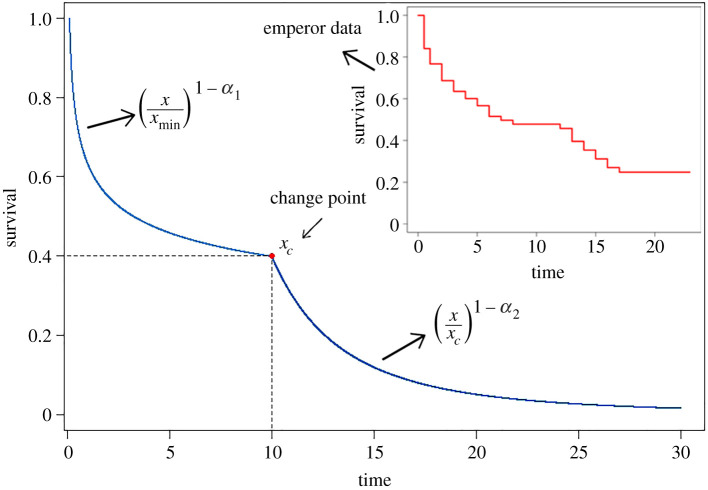
Illustration of the survival function modelled by a power-law distribution with change point *t*_*c*_. The Kaplan–Meier estimator is shown inside the figure.

To construct our model, let us consider figure 1. The top panel presents the Kaplan–Meier non-parametric estimator for the time-to-violent-death of the 69 rulers of the Roman Empire [5]. The Kaplan–Meier estimator shows the probability of an event at a certain time interval. It can be noticed that the survival time follows a power-law distribution, and after a change point, the behaviour follows a power-law distribution with a different scaling exponent. In figure 1, we consider a power-law distribution with *α*_1_ parameter and after a critical value *t*_*c*_, the behaviour change to a power-law distribution with coefficient *α*_2_.

In a general form, the power-law model behaviour in figure 1 can be represented by the following expression:

2.1 $$S(t)={\left(\frac{t}{t_{\min}}\right)}^{1-\alpha_{1}}I[t_{\min},t_{c}]+C{\left(\frac{t}{t_{c}}\right)}^{1-\alpha_{2}}I(t_{c},\infty ),$$

where I(⋅) is an identity function that returns 1 if the value is inside the interval and 0 otherwise, $C={\left(t_{c}/t_{\min}\right)}^{1-\alpha_{1}}$ is the normalized constant that make the model a complementary cumulative function and *t*_*c*_ > *t*_min_. From equation (2.1) the probability distribution of the power-law distribution with change point is given by

2.2 $$f(t)=\frac{\alpha_{1}-1}{t_{\min}}{\left(\frac{t}{t_{\min}}\right)}^{-\alpha_{1}}I[t_{\min},t_{c}]+C\frac{\alpha_{2}-1}{t_{c}}{\left(\frac{t}{t_{c}}\right)}^{-\alpha_{2}}I(t_{c},\infty ).$$

An important aspect of the proposed model is that it can be seen as a generalization of the power-law distribution in the thermodynamic limit of *t*_*c*_, i.e.

$$\lim_{t_{c}\to \infty }S(t)={\left(\frac{t}{t_{\min}}\right)}^{1-\alpha_{1}},$$

which leads to the standard power-law distribution.

In many cases, it is useful to present other mathematical functions related to the proposed model, such as the mean, variance and kurtosis, to name a few. These functions can be obtained from the *r*th moment that is derived in appendix A.1. Another aspect that needs to be discussed is the inferential procedure used to obtain the parameter estimates. Although the MLE has already been discussed for the power-law distribution (see Clauset *et al.* [14] and references therein), this method needs to be generalized to our model. The closed-form estimators for the parameters and the asymptotic confidence intervals obtained from the maximum-likelihood estimators are discussed in detail in appendix A.2.

The estimators presented in appendix A for complete data can be used satisfactorily in most scenarios. However, these estimators may return biased estimates in the presence of events with incomplete information. For instance, Titus rules for less than 1 year and died, probably owing to natural causes, however, he never experienced the event of interest, i.e. ‘a violent death’, in this case, his information is considered as censored, he may or may not experience such type of death if he had ruled for a long period such as Augustus. To take into account such information, we proposed a model that accommodates the presence of two distinct groups: one that suffered a violent death and the other that would not suffer a violent death even if we wait for a long time. These models are known as long-term survival distributions and the parameter estimation that takes into account this characteristic is discussed in detail in appendix A.2.2. This procedure is used to achieve the estimates for the datasets discussed above.

## 3. Results

We divide our research into two parts. Initially, we analyse the data by Saleh [5], which considers the western Roman Empire, from Augustus (d. 14 CE) to Theodosius (d. 395 CE). In this analysis, we compare our model with the previous model discussed in [5]. Our study includes the eastern Roman Empire, which ended with Constantine XI Palaiologos (d. 1453 CE). While the first dataset considers only 69 emperors, the second one is complete, covering 175 Roman emperors.

### 3.1. Western Roman Empire

Initially, we consider the data organized by Saleh [5], which was obtained from De Imperatoribus Romanis, an online encyclopedia of Roman emperors available as supplementary material in the cited paper. Here, we consider the same dataset in order to compare the two models. Saleh [5] has considered a mixture of Weibull distributions to describe the lifetime, given by

3.1 $${\hat{S}}_{MW}(t)=0.876\cdot \exp\left[-{\left(\frac{t}{12.835}\right)}^{0.618}\right]+0.124\cdot \exp\left[-{\left(\frac{t}{14.833}\right)}^{13.387}\right].$$

An important characteristic that should be taken into account when analysing data from the Roman Empire is the existence of emperors who did not have a violent death, i.e. a portion of the emperors was not susceptible to the event of interest. From the model (equation (3.1)), we have that *S*(*t*) → 0 as the time increases. Hence, the model assumes that all the emperors will experience a violent death at some time. This assumption is not plausible, as many emperors died owing to natural causes and did not have a violent death. Such characteristics can be incorporated into our power-law model by considering a long-term survival model. In this case, *S*(*t*) → *π* as the time increase, where *π* ≥ 0 is the proportion of emperors that did not experience a violent death. The details related to the modifications in the power-law distribution with a change point to include long-term survival can be seen in appendix A.4.

Fitting the power-law distribution to the data, the MLEs are obtained and return the estimates ${\hat{\alpha }}_{1}=1.41$, ${\hat{\alpha }}_{2}=5.12$ and *π* = 0.22 when fixing *t*_min_ = 0.5 and *t*_*c*_ = 13. Although these results returned good shapes for the survival function we observe that there is space for improvement. Considering a simple grid search around the obtained values we achieve the following estimates ${\hat{\alpha }}_{1}=1.382$, ${\hat{\alpha }}_{2}=8.5$ and *π* = 0.245 which provided better curves when compared with the empirical survival function. Hence, the adjusted model is given by

$${\hat{S}}_{PL}(t)=0.248+0.752\cdot {\left(\frac{t}{0.5}\right)}^{-0.382}I[0.5,13]+0.522\cdot {\left(\frac{t}{13}\right)}^{-7.5}I(13,\infty ).$$

**Figure 2.**
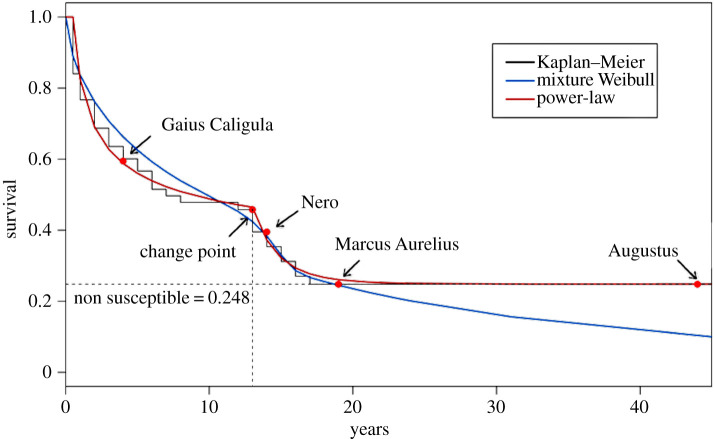
Power-law survivor (reliability) function of Roman emperors, the adjusted mixture Weibull and the nonparametric Kaplan–Meier estimates. We consider *t*_min_ = 0.5, *α*_1_ = 1.382, *t*_*c*_ = 13 and *α*_2_ = 8.5 in the power-law model shown in equation (2.1).

Owing to discontinuity in the PDF, the standard maximum likelihood of *t*_*c*_ cannot be achieved. This problem can be easily overcome by considering a grid search for the parameter where the Kolmogorov–Smirnov test is used to estimate *t*_*c*_. Figure 2 presents the fit of our proposed model compared with the mixture Weibull and the non-parametric Kaplan–Meier estimator. Additionally, we constructed a goodness-of-fit test for our proposed model (described in detail in appendix A.6) by using Geerdens *et al.* [17] method that uses Cramér–von Mises distance and a bootstrap technique to obtain the *p*-value; for the proposed dataset we obtained a *p*_value_ = 0.99, which indicates that at 5% confidence level our model can be assumed to describe the data.

Our results indicate that the change point occurs at 13 years of reign. An interesting fact is that, although Alexander the Great (d. 323 BC) has not participated in the unified Roman Empire and was not included in our analysis, his reign had 13 years. From the adjusted results, we observe that Gaius Caligula (d. 41) had a violent death before the change point, while Nero (d. 68 AC) experienced this type of death after the change point. Although we have observed about 37% of censored information, our model estimates that nearly 25% (*π* = 0.248) of the emperors would not die owing to a violent death. For example, Marcus Aurelius (d. 160 AC), known as the last of the five good emperors, lived in an age of relative peace and died owing to natural causes. As mentioned earlier, Augustus (d. 14) also reigned for 40 years and did not have a violent death. While our model can capture this effect in the survival function, the mixture Weibull distribution cannot return very far estimates when compared with the non-parametric survival, which is undesirable.

The breakpoint was observed at nearly 13 years and may have many different causes about which some speculation can be done. Saleh [5] argued that this period may be related to the mismatch between capabilities and demands under changing political and geopolitical circumstances. Christian & Elbourneb [18] discussed that low periods of rainfall led to increases in the probability that Roman troops starve, making soldiers mutiny and increasing the probability of the emperor being assassinated. Cuaresma *et al.* analysed the relationship between oil endowment and the duration of dictatorial regimes by comprising information for 106 dictators from 1875 until 2004, they observed a pattern where on average, they were in power for about 12.3 years (closer to our changing point) where 11.3% failed within the first year of their dictatorship; although the time period and the political aspects have changed significantly a similar pattern is observed in dictatorial regimes.

**Figure 3.**
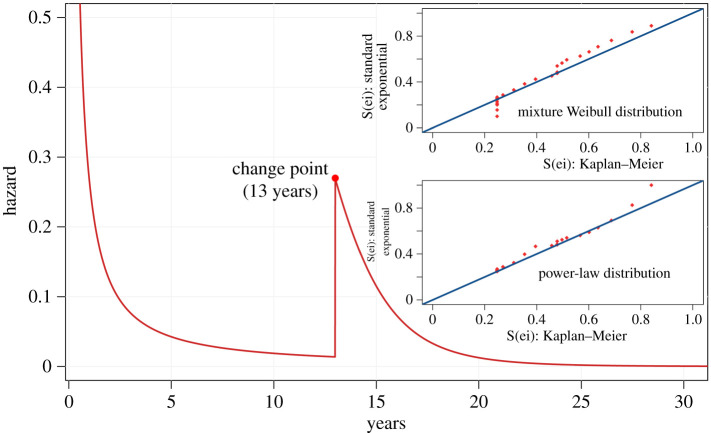
Failure rate from power-law with phase transition distribution of Roman emperors. Cox-Snell residuals analysis for the mixture Weibull and power-law distribution.

Figure 3 presents the failure rate of the adjusted model using *h*_pop_(*t*) = *f*_pop_(*t*)*S*_pop_(*t*)^−1^ (given in equations (A 9) and (A 11)) which shows a decreasing failure rate in the first years up to 13 years. During the change point, there is a rapid increase in the hazard of death (violent) and then a decrease again over time. These results differ from the ones obtained from the failure rate of the mixture Weibull distribution. In the latter, the increase in the hazard starts at nearly 11 years, reaches a peak at 15 years, and then decreases. Hence, our proposed approach returns a better understanding of the stochastic process that describes the time-to-violent-death of the emperors of the unified Roman Empire.

### 3.2. western and eastern Roman Empire

Although the analysis by Saleh [5] considered the dynasties that started from Augustus (d. 14 CE) and ended with Theodosius (d. 395 CE) when the western Roman Empire began to disintegrate, here, we extend his study considering the period of the Byzantine Empire (eastern Roman Empire) that ended with Constantine XI Palaiologos (d. 1453 CE). The additional data included 106 emperors, where the observed censorship was 56.57%, which is higher than the observed in the first analysed period, and the long-term survival was estimated in *π* = 0.404, i.e. 40.40% of the Roman emperors probably would not face a violent death. This finding implies that during the Byzantine Empire, the emperors were subjected to a less violent death. This change in the hazard behaviour may be explained owing to different causes; for instance, the eastern half of the Roman Empire was shown to be less susceptible to external attacks owing to its geography. Additionally, the Empire did not have many common boundaries with Europe. Although many other complex causes are also responsible for providing such stability, those facts combined with the internal political cohesion and more robust administrative centre allowed the Byzantine Empire to survive for many centuries.

Here, the survival function related to the time-to-violent-death for all emperors has more complex behaviour, therefore, we have generalized the power-law distribution with *k* − 1 change points that can be represented by the following expression:

3.2 $$S(t)=\sum_{i=1}^{k}{\left(\frac{t}{t_{\left(i-1\right)}^{*}}\right)}^{1-\alpha_{i}}C_{i-1}I[t_{\left(i-1\right)}^{*},t_{\left(i\right)}^{*}]\;\text{and}\;C_{\;j}=\prod_{\;j=1}^{i}{\left(\frac{t_{\left(j\right)}^{*}}{t_{\left(j-1\right)}^{*}}\right)}^{1-\alpha_{j}},$$

where I(⋅) is an identity function that returns 1 if the value is inside the interval and 0 otherwise, *C*_0_ = 1, t*_(*k*)_ = ∞, *α*_*i*_ > 0 and $t_{\left(i\right)}^{*}>t_{\left(i-1\right)}^{*},\forall i=1,\ldots ,k$. From equation (3.2) the probability distribution of the piecewise power-law model distribution is given by

3.3 $$f(t)=\sum_{i=1}^{k}\frac{\alpha_{i}-1}{t_{\left(i-1\right)}^{*}}{\left(\frac{t}{t_{\left(i-1\right)}^{*}}\right)}^{-\alpha_{i}}C_{i-1}I[t_{\left(i-1\right)}^{*},t_{\left(i\right)}^{*}].$$

**Figure 4.**
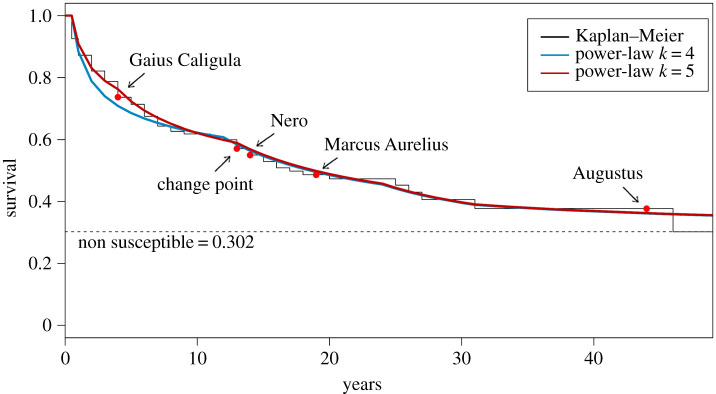
Power-law survivor (reliability) function of Roman emperors for all 175 Roman emperors and the non-parametric Kaplan–Meier estimates.

The details related to the generalized model with long-term survival and the inference procedure to estimate the parameters can be seen in appendix A. This generalization is very flexible, and the fit improves following the number of change points. The model is essentially a mixture of truncated Pareto distributions [19], although some forms of mixture have been presented earlier [20,21] they differ from our proposed model, which is not constructed based on normalized truncated Pareto distributions, in this case, the proposed approach can be seen as a piecewise power-law distribution. Additionally, there is significant literature on change point models [22–24], however, most of the results are focused on the presence of one change point in the hazard function, while other models are based on the assumption that the hazard function follows a piecewise exponential model [25,26], where the hazard rate inside each change-point are assumed to be constant, which is not the case for the proposed data. Here our aim is to show that the proposed data can be described by power-law distributions which is an important pattern that occurs in many complex systems. Figure 4 presents the survival of the adjusted model using *k* = 4, 5 as well as the presence of long-term survival.

As shown in figure 4, as the number of change points increases, we obtain a better fit for the proposed data. In fact, with *k* = 5, the adjusted model almost overlaps the non-parametric function. In order to avoid overfitting, we consider the Akaike information criteria (AIC) to discriminate between the two models. In this case, the best fit among the chosen models returns the minimum value of AIC. While the model with *k* = 4 returned an AIC = 594.10, the model with *k* = 5 yields AIC = 586.25. Thus, this is the best model for describing the time-to-violent-death for all emperors. The goodness-of-fit test was also applied for the model which for the extended dataset we obtained a *p*_value_ = 0.337, which indicates that at 5% confidence level our model can be assumed to describe the data.

### 3.3. The time-to-violent-death of western emperors

**Figure 5.**
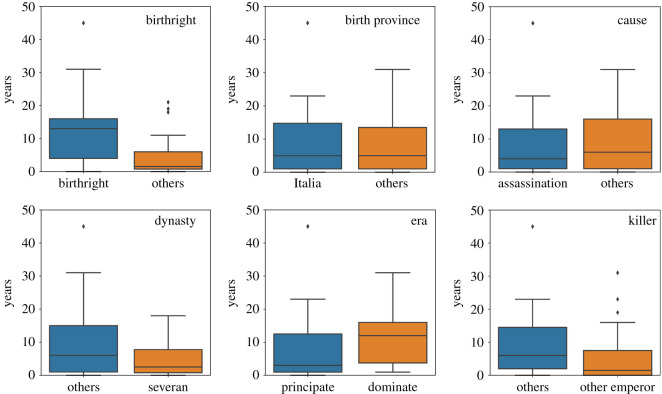
Box plot of emperors’ attributes according to the time-to-death.

We also analyse the influence of some emperors’ attributes on the time-to-violent-death. These attributes are compiled from Wikipedia and previous works, including [3,27], covering western emperors from 26 BC to 395 AD. In the case of the method of accession to power (e.g. birthright, seized power, appointment by the senate, among others), we can see in figure 5 that emperors who came from birthright tend to rule longer than from other methods. To verify the significance of this difference, we consider a two-sided test for the null hypothesis that the average time-to-violent-death is independent of the method of accession to power. If the *p*-value is smaller than the threshold, e.g. 1% or 5%, then we reject the null hypothesis of equal averages. From this hypothesis test, we conclude that the difference between the time-to-violent-death of emperors that used different methods of accession to power is significant because *p* < 0.001. This result suggests that emperors who inherited the reign tend to have a more peaceful administration and an increased probability of having a natural death. On the other hand, in terms of the birth province, there is no difference between the time-to-violent-death of emperors from Italy or other parts of the world, like Syria or Spain (*p* = 0.54). This finding indicates that Rome was very cosmopolitan, as the Empire was extensive, including many different cultures, traditions and faiths. Thus, it is expected that the place of birth plays a small part in the time-to-violent-death.

The cause of death also does not influence the time-to-violent-death. Indeed, emperors, who died owing to assassination, did not have shorter ruling periods (*p* = 0.8), although almost 40% of the emperors were assassinated. Related to this, emperors killed by other emperors also did not have a shorter reign period (*p* = 0.4), as shown in the figure.

In terms of era, we verify the presence of a moderate difference (*p* = 0.08) in terms of the reign period between the Principate. That is, the first period of the Roman Empire from the beginning of Augustus’s reign in 27 BC, and Dominate, which started after the Crisis of the Third Century, in AD 284. The reigning period is more extended during the Dominate era, with an average time of ruling of 11 years, contrasting with 7 years, verified in the Principate period covered in our dataset.

Regarding the dynasty, we also do not observe a significant difference between them in terms of the reign period (*p* = 0.2). In figure 5, we show the comparison between the Severan dynasty, which started with Septimius Severus, who rose to power as the victor of the Civil War of 193–197, and other dynasties. Although the Severan dynasty was disturbed by highly unstable family relationships and constant political turmoil [27], it did not significantly affect the time-to-violent-death.

Another important tool to analyse such data is using the Cox proportional hazards model [28], in this case, we can verify if the attributes affect the risk of death in a multiplicative way or provide higher risks during a specific time. The proportional assumption is checked using a test based on Schoenfeld residuals [29] where we present the *p*-values, assuming the threshold of 5% there is an indication of a violation of the proportional hazard assumption when the values are smaller than 0.05. The analysed attributes and its respective *p*-values in parenthesis are: birthright (0.2371), birth province (0.5000), cause (0.8785), dynasty (0.7429), era (0.2452), killer (0.0386), global (0.2554). Hence, the *p*-values indicate that only the attribute killer was shown to influence the change in the risk of death, while other covariates were shown to have proportional hazard rate differing only in a multiplicative term in the hazard function. The emperors who died by other emperors showed a higher risk of death in the beginning of their reign, on the other hand, after 16 years the curves of risks crossed each other, exhibiting a higher risk of dying by others instead of emperors. Overall, most of the attributes considered in our analysis do not affect the time-to-violent-death, which suggests that other features, like those related to external factors like wars and political conflicts, should be considered to infer the causes of the short reigning period of Roman emperors. This understanding is an exciting topic for further research.

## 4. Discussion

In this paper, we introduce a new survival model with *k* change points and power-law distribution. The model is a modification of the traditional power-law model, in which we have included the adjustments to address the long-term survival and the new procedures to obtain the parameter estimates. The model has been developed to predict the reign period of Roman emperors, motivated by the analysis provided by Saleh [5].

In examining the time-to-violent-death of the emperors of the Roman Empire, we have observed that the risk is high when the emperor assumes the throne. This finding may be related to the struggles in handling the demands that the position requires and the lack of political skills of the new emperor. Our model suggests that the risk systematically decreases until 13 years of rule and then rapidly increases after this change point. The are many possible reasons for the change in the behaviour of the risk, for instance, their old enemies had regrouped, or new ones emerged [5]. After the change point, the risk decreases again. Furthermore, the adjusted survival functions lead to 25% of emperors that would not die owing to violent death. Therefore, being a Roman emperor was a hazardous occupation that led to three out of four suffering from a cruel death during the western Empire. Additionally, taking into consideration the eastern Roman Empire, we observed that out of three emperors suffer from a cruel death.

The comparison with the model by Saleh [5] shows that the power-law model is more accurate to describe the variable of interest, i.e. the time-to-violent-death of the emperors of the Roman Empire. This fact has important implications since the power-law distribution is ubiquitous in natural and artificial systems and is often related to critical events. Power laws can be observed in earthquakes [7–9], in solar radiophysics [10], stock price fluctuations [11] and tree-limb branching [12]. In the dataset considered here, the lifetime of Roman emperors decay as a power law, which implies that short reigns are common, while high occurrences are rare. This behaviour is similar to that observed in many systems, including earthquake, where huge earthquakes are rare. This result suggests that the time-to-violent-death does not have a typical scale, including the Roman Empire as an example of systems presenting scale invariances, such as the distribution of income, size of cities according to population, size of corporations and word frequencies [14].

Our power-law survival model with *k* change points is general and can be applied to model the hazard rate function in engineering, medicine, economy, and physics, especially in situations where we have a good indication that the tail of the distribution follows a power law. However, the earlier occurrences may have a more slow decay. In addition to the proposed results drawn here, there are additional aspects of the new distribution that should be considered. For instance, the theoretical results assuming discrete data are currently under investigation. Under this scenario, the estimation procedures do not have a closed-form expression and need to be further improved. Thus, our results open several possibilities in applications and theoretical development, including models with cut-off and improvements by considering Bayesian methods.

## Figures and Tables

**Figure 6.**
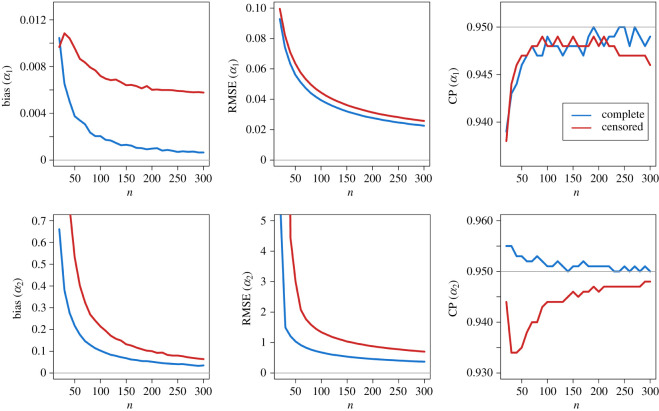
Bias and RMSEs for *α*_1_ and *α*_2_ considering *t*_*m*_ = 0.5, *t*_*c*_ = 13, *α*_1_ = 1.3 and *α*_2_ = 6 for *N* = 200 000 random samples and *n* = (20, 60, …, 300).

## Data accessibility

The dataset used is presented in the electronic supplementary material [31].

## Appendix A. Methods

This section contains most of the technical information related to the proposed model. We will discuss the mathematical properties such as the higher moments which allow us to obtain the mean, variance, among other measures. We also discuss the inferential procures to obtain the parameter estimates under the maximum-likelihood estimators in the presence of complete and censored data.

**A.1. Mathematical properties**

The *r*th moment is a useful function that can be used to obtain different measures related to the proposed distributions, including the mean, variance and kurtosis. For the proposed distribution, the *r*th moment is given by

A 1 $$\begin{array}{rl} \mu_{r} & =\int_{t_{\min}}^{\infty }t^{r}f(t)\;dt=\int_{t_{\min}}^{t_{c}}t^{r}\frac{\alpha_{1}-1}{t_{\min}}{\left(\frac{t}{t_{\min}}\right)}^{-\alpha_{1}}\;dt+\int_{t_{c}}^{\infty }C\frac{\alpha_{2}-1}{t_{c}}{\left(\frac{t}{t_{c}}\right)}^{-\alpha_{2}}\;dt\\ & =\frac{\left(t_{c}^{r-\alpha_{1}+1}-t_{\min}^{r-\alpha_{1}+1})(\alpha_{1}-1\right)}{(r-\alpha_{1}+1)t_{\min}^{1-\alpha_{1}}}+\frac{(\alpha_{2}-1)t_{c}^{r-\alpha_{1}+1}}{(r-\alpha_{2}+1)t_{\min}^{1-\alpha_{1}}}, \end{array}$$

where *r* < *α* − 1.

Because the proposed distribution is an extension of the power-law distribution, when *t*_*c*_ → ∞ we have

$$\lim_{t_{c}\to \infty }\frac{(\alpha_{2}-1)t_{c}^{r-\alpha_{1}+1}}{(r-\alpha_{2}+1)t_{\min}^{1-\alpha_{1}}}=0.$$

Additionally, applying the same limit in the left side of the *r*th moment we have

$$\lim_{t_{c}\to \infty }\frac{\left(t_{c}^{r-\alpha_{1}+1}-t_{\min}^{r-\alpha_{1}+1})(\alpha_{1}-1\right)}{(r-\alpha_{1}+1)t_{\min}^{1-\alpha_{1}}}=\frac{\left(\alpha_{1}-1\right)}{\left(\alpha_{1}-r-1\right)}t_{\min}^{r}.$$

Hence, if *t*_*c*_ → ∞ we have that $\mu_{r}=(\left(\alpha_{1}-1\right)/\left(\alpha_{1}-r-1\right))t_{\min}^{r}$ which is the *r*th moment of the standard power-law distribution.

The mean and variance are easily obtained from equation (A 1) by considering that *μ* = *μ*_1_ and $\mathrm{Var}(X)=\mu_{1}^{2}-\mu_{2}$. Additionally, measures such as skewness and kurtosis can also be obtained in closed-form using the higher orders of the obtained moments.

The hazard function describe the instantaneous risk of failure at a given of *t* is given by

A 2 $$h(t)=\frac{\alpha_{1}-1}{t_{\min}}{\left(\frac{t}{t_{\min}}\right)}^{-1}I[t_{\min},t_{c}]+\frac{\alpha_{2}-1}{t_{c}}{\left(\frac{t}{t_{c}}\right)}^{-1}I(t_{c},\infty ).$$

The hazard function may exhibit different forms such as increasing, decreasing, unimodal, bathtub behaviour among others. In this case, (*t*/*t*_min_)^−1^ is an decreasing function multiplied by a constant term, hence, the hazard function initially has decreasing shape until *t*_*c*_. Additionally, because (*t*_*c*_/*t*_min_)^−1^ < (*t*_*c*_/*t*_*c*_)^−1^ = 1, the risk increases rapidly and then decreases again as (*t*/*t*_*c*_)^−1^ is a decreasing function (see for instance figure 3).

**A.2. Inference for the parameters**

Usually, the parameters associated with the introduced distribution are unknown and need to be estimated. There are different estimation methods proposed in the literature. In this work, we have considered the maximum-likelihood method. In this section, we discuss how to calculate the estimates assuming complete data. Further, we present the MLEs in the presence of random censoring, a general type of censoring that occurs in many situations.

**A.2.1. Maximum-likelihood estimator**

The maximum-likelihood method has been widely used owing to its good asymptotic properties. The estimates are obtained from the maximization of the likelihood function given by

$$\begin{array}{rl} L(\alpha_{1},\alpha_{2}|t_{\min},t_{c},t) & =\prod_{i=1}^{n}\left(\frac{\alpha_{1}-1}{t_{\min}}{\left(\frac{t_{i}}{t_{\min}}\right)}^{-\alpha_{1}}I[t_{\min},t_{c}]+C\frac{\alpha_{2}-1}{t_{c}}{\left(\frac{t_{i}}{t_{c}}\right)}^{-\alpha_{2}}I(t_{c},\infty )\right)\\ & =\prod_{i\;:\;t_{i}<t_{c}}\left(\frac{\alpha_{1}-1}{t_{\min}}{\left(\frac{t}{t_{\min}}\right)}^{-\alpha_{1}}\right)\prod_{i\;:\;t_{i}\geq t_{c}}\left(C\frac{\alpha_{2}-1}{t_{c}}{\left(\frac{t}{t_{c}}\right)}^{-\alpha_{2}}\right). \end{array}$$

Instead of direct maximizing the likelihood function, it is straightforward to maximize the log-likelihood function that is given by

$$\begin{array}{rl} & l(\alpha_{1},\alpha_{2}|t_{\min},t_{c},t)=n_{1}\log(\alpha_{1}-1)-n_{1}(1-\alpha_{1})\log(t_{\min})-\alpha_{1}\sum_{i\;:\;t_{i}<t_{c}}\;\log(t_{i})+n_{2}\log(\alpha_{2}-1)\\ & \;-n_{2}(\alpha_{1}-\alpha_{2})\log(t_{c})-n_{2}(1-\alpha_{1})\log(t_{\min})-\alpha_{2}\sum_{i\;:\;t_{i}\geq t_{c}}\log(t_{i}). \end{array}$$

From the expressions (∂/∂*α*_1_)*l*(*α*_1_, *α*_2_|*t*_min_, *t*_*c*_, *t*) = 0, (∂/∂*α*_2_)*l*(*α*_1_, *α*_2_|*t*_min_, *t*_*c*_, *t*) = 0, the likelihood equations are given as

A 3 $${\hat{\alpha }}_{1}=1+n_{1}{\left(\sum_{i\;:\;t_{i}<t_{c}}\log\left(\frac{t_{i}}{t_{\min}}\right)+n_{2}\log\left(\frac{t_{c}}{t_{\min}}\right)\right)}^{-1}$$

and

A 4 $${\hat{\alpha }}_{2}=1+n_{2}{\left(\sum_{i\;:\;t_{i}\geq t_{c}}\log\left(\frac{t_{i}}{t_{c}}\right)\right)}^{-1}.$$

The MLE is asymptotically normal distributed with a normal distribution given by $({\hat{\alpha }}_{1},{\hat{\alpha }}_{2})∼N((\alpha_{1},\alpha_{2}),I^{-1}(\alpha_{1},\alpha_{2})))\text{ for }n\to \infty$, where *I*^−1^(*α*_1_, *α*_2_) is the Fisher information matrix with elements given by

A 5 $$I_{11}^{-1}(\alpha_{1},\alpha_{2})=-E\left[\frac{\partial^{2}}{\partial \alpha^{2}}l(\alpha ;t)\right]=\frac{{\left(\alpha_{1}-1\right)}^{2}}{n_{1}},\;I_{22}^{-1}(\alpha_{1},\alpha_{2})=-E\left[\frac{\partial^{2}}{\partial \alpha^{2}}l(\alpha ;t)\right]=\frac{{\left(\alpha_{2}-1\right)}^{2}}{n_{2}}$$

and $I_{12}^{-1}(\alpha )=I_{21}^{-1}(\alpha )=0$.

**A.2.2. The presence of random censoring**

In the presence of partial information the likelihood function needs to be modified. Let *T*_*i*_ be the lifetime of the *i*th emperor with censoring time *C*_*i*_, which are assumed to be independent of *T*_*i*_s and its distribution does not depend on the parameters, the dataset is represented by $D=(t_{i},\delta_{i})$, where *t*_*i*_ = min (*T*_*i*_, *C*_*i*_) and *δ*_*i*_ = *I*(*T*_*i*_ ≤ *C*_*i*_). The likelihood function for θ is given by

$$\begin{array}{rl} L(\alpha_{1},\alpha_{2};t_{\min},t_{c},t,\delta ) & =\prod_{i=1}^{n}f{\left(t_{i}|\theta \right)}^{\delta_{i}}S{\left(t_{i}|\theta \right)}^{1-\delta_{i}}\\ & =\prod_{i\;:\;t_{i}<t_{c}}\;\left(\frac{\alpha_{1}-1}{t_{\min}}{\left(\frac{t}{t_{\min}}\right)}^{1-\delta_{i}-\alpha_{1}}\right)\prod_{i\;:\;t_{i}\geq t_{c}}\;\left(C\frac{\alpha_{2}-1}{t_{c}}{\left(\frac{t}{t_{c}}\right)}^{1-\delta_{i}-\alpha_{2}}\right). \end{array}$$

The log-likelihood function is given by

$$\begin{array}{rl} & l(\alpha_{1},\alpha_{2}|t_{\min},t_{c},t)=d_{1}\log(\alpha_{1}-1)-d_{1}(1-\alpha_{1})\log(t_{\min})+\sum_{i\;:\;t_{i}<t_{c}}(1-\delta_{i}-\alpha_{1})\log(t_{i})\\ & \;+d_{2}\log(\alpha_{2}-1)+n_{2}(1-\alpha_{1})\log\left(\frac{t_{c}}{t_{\min}}\right)-d_{2}\log(t_{c})+\sum_{i\;:\;t_{i}\geq t_{c}}(1-\delta_{i}-\alpha_{2})\log\left(\frac{t_{i}}{t_{c}}\right). \end{array}$$

From the expressions (∂/∂*α*_1_)*l*(*α*_1_, *α*_2_|*t*_min_, *t*_*c*_, *t*) = 0, (∂/∂*α*_2_)*l*(*α*_1_, *α*_2_|*t*_min_, *t*_*c*_, *t*) = 0, the likelihood equations are given as

A 6 $${\hat{\alpha }}_{1}=1+d_{1}{\left(\sum_{i\;:\;t_{i}<t_{c}}\log\left(\frac{t_{i}}{t_{\min}}\right)+n_{2}\log\left(\frac{t_{c}}{t_{\min}}\right)\right)}^{-1}$$

and

A 7 $${\hat{\alpha }}_{2}=1+d_{2}{\left(\sum_{i\;:\;t_{i}\geq t_{c}}\log\left(\frac{t_{i}}{t_{c}}\right)\right)}^{-1}.$$

Similar to the case of complete data the MLE is asymptotically normal distributed with a normal distribution given by $({\hat{\alpha }}_{1},{\hat{\alpha }}_{2})∼N((\alpha_{1},\alpha_{2}),I^{-1}(\alpha_{1},\alpha_{2})))\text{ for }n\to \infty$, where *I*^−1^(*α*_1_, *α*_2_) is the Fisher information matrix with elements given by

$$I_{11}^{-1}(\alpha_{1},\alpha_{2})=-E\left[\frac{\partial^{2}}{\partial \alpha^{2}}l(\alpha ;t)\right]=\frac{{\left(\alpha_{1}-1\right)}^{2}}{d_{1}},\;I_{22}^{-1}(\alpha_{1},\alpha_{2})=-E\left[\frac{\partial^{2}}{\partial \alpha^{2}}l(\alpha ;t)\right]=\frac{{\left(\alpha_{2}-1\right)}^{2}}{d_{2}}$$

and $I_{12}^{-1}(\alpha )=I_{21}^{-1}(\alpha )=0$.

**A.3. Simulation analysis**

This section is devoted to comparing the quality of our proposed approach in terms of minimum bias and the mean-square error (MSE). The metrics are computed by

$${\mathrm{Bias}}_{\alpha }=\frac{1}{N}\sum_{i=1}^{N}({\hat{\alpha }}_{i}-\alpha )\;\text{and}\;{\mathrm{MSE}}_{\alpha }=\sqrt{\sum_{i=1}^{N}\frac{{\left({\hat{\alpha }}_{i}-\alpha \right)}^{2}}{N}},$$

where *N* = 200 000 is the number of estimates obtained through the MLE. An adequate estimation method will return the bias and the MSEs closer to zero. The software R (R Core Development Team) was used to conduct this simulation study. The codes are available in the electronic supplementary material.

The pseudo-random samples from a power-law distribution with change phase can be easily generated by considering the quartile function that is given by

A 8 $$t_{u}=\left\{ \begin{array}{cc} t_{m}{\left(1-u\right)}^{1/(1-\alpha_{1})} & \mathrm{if}\;u\geq (1-c)\\ t_{c}{\left(\frac{1-u}{c}\right)}^{1/(1-\alpha_{2})} & \mathrm{if}\;u>(1-c), \end{array}\right.$$

where *u* is generated from a uniform distribution with range (0,1). Hence, setting the parameters and repeating the process *n* times we obtain a pseudo-random sample of size *n* that is considered as complete data. To generate censored data an additional step is necessary. Because we have assumed that the censoring times *C*_*i*_ are assumed to be independent of *X*_*i*_s and we sample a new value *y* from a Uniform(0, *y*_max_), where *y*_max_ is the value that allows us to obtain the desirable proportion of censoring, then the observed time is the minimum (*t*_*u*_, *y*) and it is considered complete if the value is *t*_*u*_ or censored if the value came from *y*. Here, we assume that the values are *t*_*m*_ = 0.5, *t*_*c*_ = 13, *α*_1_ = 1.3 and *α*_2_ = 6 and *y*_max_ is selected to obtain on average the proportion of censoring of 0.37. The upper bound distribution in figure 6 displays the bias and the root MSEs (RMSEs) from the estimates of *α* obtained using the MC method. The horizontal lines in the figures correspond to bias and RMSEs being one and zero, respectively.

As shown in figure 6, the results related to *α*_1_ do not have large values, it can be observed that the bias is minimal for both complete and censored data, while *α*_2_ imposes more bias in the presence of small size samples. On the other hand, both bias and RMSEs tend to zero when *n* increases. Moreover, we obtain accurate coverage probability through the confidence intervals, because they cover the true values for the parameters even for small samples. Overall, these results show that the MLE estimators could be considered for estimating the parameters of the proposed model.

**A.4. A long-term survivor power-law distribution**

Here we suppose that the population is split into two groups: those emperors that are not susceptible to the event of interest with probability *π*, and those who are susceptible (in risk) to the event with probability (1 − *π*). The event of interest is ‘a violent death’. The long-term survival function is then given by

A 9 $$S_{\mathrm{pop}}(t)=\pi +(1-\pi )S_{0}(t),$$

where *π* ∈ (0, 1) and *S*_0_(*t*) denotes the baseline survival function for the susceptible group in the population.

This model is known as the standard long-term survival model and was introduced by Berkson & Gage [30]. Assuming that the baseline survival function is given by our model, the obtained (unconditional) survival function (A 9) is given by

A 10 $$S_{\mathrm{pop}}(t)=\pi +(1-\pi ){\left(\frac{t}{t_{\min}}\right)}^{1-\alpha_{1}}I[t_{\min},t_{c}]+(1-\pi )C{\left(\frac{t}{t_{c}}\right)}^{1-\alpha_{2}}I(t_{c},\infty ).$$

From the equation above, we can obtain its related density function that is given by

A 11 $$f_{\mathrm{pop}}(t)=(1-\pi )\frac{\alpha_{1}-1}{t_{\min}}{\left(\frac{t}{t_{\min}}\right)}^{-\alpha_{1}}I[t_{\min},t_{c}]+(1-\pi )C\frac{\alpha_{2}-1}{t_{c}}{\left(\frac{t}{t_{c}}\right)}^{-\alpha_{2}}I(t_{c},\infty ).$$

Here, we have included an additional parameter of *π* that needs to be estimated. Under the MLEs and assuming that the data have random censoring, the estimates are obtained from the maximization of the likelihood function are given by

$$\begin{array}{rl} & L(\alpha_{1},\alpha_{2},\pi ;t_{\min},t_{c},t,\delta )=\prod_{i=1}^{n}f{\left(t_{i}|\theta \right)}^{\delta_{i}}S{\left(t_{i}|\theta \right)}^{1-\delta_{i}}\\ & =\prod_{i\;:\;t_{i}<t_{c}}{\left((1-\pi )\frac{\alpha_{1}-1}{t_{\min}}{\left(\frac{t}{t_{\min}}\right)}^{-\alpha_{1}}\right)}^{\delta_{i}}{\left(\pi +(1-\pi ){\left(\frac{t}{t_{\min}}\right)}^{1-\alpha_{1}}\right)}^{1-\delta_{i}}\\ & \times \prod_{i\;:\;t_{i}\geq t_{c}}{\left((1-\pi )C\frac{\alpha_{2}-1}{t_{c}}{\left(\frac{t}{t_{c}}\right)}^{-\alpha_{2}}\right)}^{\delta_{i}}{\left(\pi +(1-\pi )C{\left(\frac{t}{t_{c}}\right)}^{1-\alpha_{2}}\right)}^{1-\delta_{i}}. \end{array}$$

The log-likelihood function that is used to find the estimates is given by

$$\begin{array}{rl} l(\alpha_{1},\alpha_{2},\pi ;t_{\min},t_{c},t,\delta ) & =\sum_{i\;:\;t_{i}<t_{c}}\delta_{i}\log\left((1-\pi )\frac{\alpha_{1}-1}{t_{\min}}{\left(\frac{t}{t_{\min}}\right)}^{-\alpha_{1}}\right)\\ & \;+\sum_{i\;:\;t_{i}<t_{c}}(1-\delta_{i})\log\left(\pi +(1-\pi ){\left(\frac{t}{t_{\min}}\right)}^{1-\alpha_{1}}\right)\\ & \;+\sum_{i\;:\;t_{i}\geq t_{c}}\delta_{i}\log\left((1-\pi )C\frac{\alpha_{2}-1}{t_{c}}{\left(\frac{t}{t_{c}}\right)}^{-\alpha_{2}}\right)\\ & \;+\sum_{i\;:\;t_{i}<t_{c}}(1-\delta_{i})\log\left(\pi +(1-\pi )C{\left(\frac{t}{t_{c}}\right)}^{1-\alpha_{2}}\right). \end{array}$$

The MLEs are obtained by solving the expressions (∂/∂*α*_1_)*l*(*α*_1_, *α*_2_, *π*; *t*_min_, *t*_*c*_, ***t, δ***) = 0, (∂/∂*α*_2_)*l*(*α*_1_, *α*_2_, *π*; *t*_min_, *t*_*c*_, ***t, δ***) = 0 and (∂/∂*π*)*l*(*α*_1_, *α*_2_, *π*; *t*_min_, *t*_*c*_, ***t, δ***) = 0. Unfortunately for this scenario, the MLEs do not have closed-form expression and numerical methods such as Newton–Raphson need to be used to find the solution of these nonlinear equations.

**A.5. Long-term power-law model with *k* − 1 change points**

Assuming that the baseline survival function is given by power-law distribution with *k* − 1 change points, the related long-term survival model is given by

A 12 $$S_{\mathrm{pop}}(t)=\pi +(1-\pi )\sum_{i=1}^{k}{\left(\frac{t}{t_{\left(i-1\right)}^{*}}\right)}^{1-\alpha_{i}}C_{i-1}I[t_{\left(i-1\right)}^{*},t_{\left(i\right)}^{*}].$$

From the equation above, we can obtain its related density function that is given by

A 13 $$f_{\mathrm{pop}}(t)=(1-\pi )\sum_{i=1}^{k}\frac{\alpha_{i}-1}{t_{\left(i-1\right)}^{*}}{\left(\frac{t}{t_{\left(i-1\right)}^{*}}\right)}^{-\alpha_{i}}C_{i-1}I[t_{\left(i-1\right)}^{*},t_{\left(i\right)}^{*}].$$

Under the MLEs and assuming that the data have random censoring, the estimates are obtained from the maximization of the likelihood function given by

$$\begin{array}{rl} L(\Theta ,t_{c},t,\delta ) & =\prod_{i=1}^{n}{\left((1-\pi )\sum_{\;j=1}^{k}\frac{\alpha_{j}-1}{t_{\left(j-1\right)}^{*}}{\left(\frac{t_{i}}{t_{\left(j-1\right)}^{*}}\right)}^{-\alpha_{j}}C_{\;j-1}I[t_{\left(j-1\right)}^{*},t_{\left(j\right)}^{*}]\right)}^{\delta_{i}}\\ & \times {\left(\pi +(1-\pi )\sum_{i=1}^{k}{\left(\frac{t_{i}}{t_{\left(j-1\right)}^{*}}\right)}^{1-\alpha_{j}}C_{\;j-1}I[t_{\left(j-1\right)}^{*},t_{\left(j\right)}^{*}]\right)}^{1-\delta_{i}}\\ & =\prod_{\;j=1}^{k}[\prod_{i\;:\;t_{i}\in [t_{\left(j-1\right)}^{*},t_{\left(j\right)}^{*}]}{\left((1-\pi )C_{\;j-1}\left(\frac{\alpha_{j}-1}{t_{\left(j-1\right)}^{*}}{\left(\frac{t_{i}}{t_{\left(j-1\right)}^{*}}\right)}^{-\alpha_{j}}\right)\right)}^{\delta_{i}}\\ & \times {\left(\pi +(1-\pi ){\left(\frac{t_{i}}{t_{\left(j-1\right)}^{*}}\right)}^{1-\alpha_{j}}C_{\;j-1}\right)}^{1-\delta_{i}}]. \end{array}$$

The log-likelihood function that is used to find the estimates is given by

$$\begin{array}{rl} l(\Theta ,t_{c},t,\delta )= & \sum_{\;j=1}^{k}\left[\sum_{i\;:\;t_{i}\in [t_{\left(j-1\right)}^{*},t_{\left(j\right)}^{*}]}\delta_{i}\log\left((1-\pi )C_{\;j-1}\left(\frac{\alpha_{j}-1}{t_{\left(j-1\right)}^{*}}{\left(\frac{t_{i}}{t_{\left(j-1\right)}^{*}}\right)}^{-\alpha_{j}}\right)\right)\right]\\ & \;+\sum_{\;j=1}^{k}\left[\sum_{i\;:\;t_{i}\in [t_{\left(j-1\right)}^{*},t_{\left(j\right)}^{*}]}(1-\delta_{i})\log\left(\pi +(1-\pi ){\left(\frac{t_{i}}{t_{\left(j-1\right)}^{*}}\right)}^{1-\alpha_{j}}C_{\;j-1}\right)\right]. \end{array}$$

**A.6. Goodness-of-fit test for censoring and long-term survival**

Here, we adapt the procedures developed by Geerdens *et al.* [17] which comprehends in a goodness-of-fit test for a selected model in the presence of censored data and long-term survival. Let us recall the used notations where *T*_*i*_ is the lifetime of the *i*th emperor with censoring time *C*_*i*_, which are assumed to be independent of *T*_*i*_s and its distribution does not depend on the parameters, where *t*_*i*_ = min (*T*_*i*_, *C*_*i*_) and *δ*_*i*_ = *I*(*T*_*i*_ ≤ *C*_*i*_). Additionally, consider that

$$\hat{\phi }=1-\hat{S}(\max\left(t_{i}|\delta_{i}=1\right))\;\text{and}\;{\hat{S}}_{1}(t)=\frac{\hat{\phi }-1+\hat{S}(t)}{\hat{\phi }},$$

where $\hat{\phi }$ is the non-parametric estimator of *ϕ* and $\hat{S}(t)$ is the Kaplan–Meier estimator of *S*(*t*). Let $S_{1,\hat{\theta }}(t)$ where *θ* is the vector of parameters in the assumed parametric form of *S*_1_(*t*). The statistic based on the Cramér–von Mises distance is given by

A 14 $$\Lambda_{n}=\sum_{i=1}^{n}({\hat{S}}_{1}(t)-S_{1,\hat{\theta }}{\left(t)\right)}^{2}.$$

In this case, the hypotheses is

$$H_{0}\;:\;S_{1}\in \{S_{1,\theta }\;:\;\theta \in \Theta \}\;\mathrm{versus}\;H_{1}\;:\;S_{1}\notin \{S_{1,\theta }\;:\;\theta \in \Theta \},$$

where Θ is the parametric space of *θ*. The authors derived the asymptotic distribution of the test statistic, however, they proposed a bootstrap approach to be used for small and moderate samples. The algorithm is described below:

(i) under $H_{0}$, compute the estimates $\hat{\phi }$, $\hat{\theta }$ and the test statistic $\Lambda_{n}$ presented in (A 14),
(ii) generate *b* = 1, …, *B* resamples based on the steps above,
(iii) generate $Y_{i}^{b}$ where $Y_{i}^{b}∼\text{Be}(\hat{\phi }),i=1,\ldots ,n$,
(iv) sample $T_{i}^{b}$ from $S_{1,\hat{\theta }}(\cdot )$ if $Y_{i}^{b}=1$ or set $T_{i}^{b}=\infty$ if $Y_{i}^{b}=0$,
(v) generate $C_{i}^{b}$ from $1-\hat{G}(\cdot )$, the KM estimator of 1 − *G*( · ),
(vi) set $T_{i}^{b}=\text{min}(T_{i}^{b},C_{i}^{b})$ and $\delta_{i}^{b}=I(T_{i}^{b}\leq C_{i}^{b})$,
(vii) compute the test statistic for the *b* sample:
  $$\Lambda_{n}^{b}=\sum_{i=1}^{n}{\left({\hat{S}}_{1}^{b}(T_{i}^{b})-S_{1,{\hat{\theta }}^{b}}(T_{i}^{b})\right)}^{2},$$
  where ${\hat{S}}_{1}^{b}(\cdot )$ is the KM estimator of ${\hat{S}}_{1}$ while ${\hat{\theta }}^{b}$ is the MLE of *θ*^*b*^ based on the bootstrap sample, and
(viii) finally, compute the approximate *p*-value from $p_{\mathrm{boot}}=1/B\sum_{b=1}^{B}I(\Lambda_{n}^{b}\geq \Lambda_{n})$.

We assume that the parametric form is given by the long-term power-law model with *k* − 1 change points and the number of bootstrap replications are *B* = 1000.
