# Supplementary material for: Power laws in the Roman Empire: a survival analysis
Source: R Soc Open Sci. 2021 Jul 28;8(7):210850. doi: 10.1098/rsos.210850 (PMC8316818; doi:10.1098/rsos.210850)
Supplement: Supplementary file 1 [file rsos210850_review_history.pdf]

THE ROYAL SOCIETY
PUBLISHING

ROYAL SOCIETY
OPEN SCIENCE

Power laws in the Roman Empire: a survival analysis

P.L. Ramos, L. F. Costa, F. Louzada and F. A. Rodrigues

Article citation details
R. Soc. open sci. **8**: 210850.
http://dx.doi.org/10.1098/rsos.210850

Review timeline
Original submission: 26 May 2021
Final acceptance: 21 June 2021

Note: Reports are unedited and appear as submitted by the referee. The review history appears in chronological order.

Note: This manuscript was transferred from another Royal Society journal with peer review.

Review History

Decision letter (RSOS-210850.R0)

We hope you are keeping well at this difficult and unusual time. We continue to value your support of the journal in these challenging circumstances. If Royal Society Open Science can assist you at all, please don't hesitate to let us know at the email address below.

Dear Dr Rodrigues:

I am pleased to inform you that your manuscript entitled "Power laws in the Roman Empire: a survival analysis" is now accepted for publication in Royal Society Open Science.

Please ensure that you send to the editorial office an editable version of your accepted manuscript, and individual files for each figure and table included in your manuscript. You can send these in a zip folder if more convenient. Failure to provide these files may delay the processing of your proof. You may disregard this request if you have already provided these files to the editorial office.

If you have not already done so, please remember to make any data sets or code libraries 'live' prior to publication, and update any links as needed when you receive a proof to check - for instance, from a private 'for review' URL to a publicly accessible 'for publication' URL. It is good practice to also add data sets, code and other digital materials to your reference list.

Reports © 2021 The Reviewers; Decision Letters © 2021 The Reviewers and Editors; Responses © 2021 The Reviewers, Editors and Authors. Published by the Royal Society under the terms of the Creative Commons Attribution License http://creativecommons.org/licenses/by/4.0/, which permits unrestricted use, provided the original author and source are credited

You can expect to receive a proof of your article in the near future. Please contact the editorial office (openscience@royalsociety.org) and the production office (openscience_proofs@royalsociety.org) to let us know if you are likely to be away from e-mail contact -- if you are going to be away, please nominate a co-author (if available) to manage the proofing process, and ensure they are copied into your email to the journal. Due to rapid publication and an extremely tight schedule, if comments are not received, your paper may experience a delay in publication.

Please see the Royal Society Publishing guidance on how you may share your accepted author manuscript at https://royalsociety.org/journals/ethics-policies/media-embargo/. After publication, some additional ways to effectively promote your article can also be found here https://royalsociety.org/blog/2020/07/promoting-your-latest-paper-and-tracking-your-results/.

On behalf of the Editors of Royal Society Open Science, thank you for your support of the journal and we look forward to your continued contributions to Royal Society Open Science.

Kind regards,
Royal Society Open Science Editorial Office
Royal Society Open Science
openscience@royalsociety.org

on behalf of Dr Robert MacKay (Associate Editor) and Professor Mark Chaplain (Subject Editor).

Associate Editor Dr Robert MacKay Comments to Author:

Associate Editor
Comments to the Author:
The reviewers comments on a previous version appear to have been adequately addressed, so I recommend publication.

Follow Royal Society Publishing on Twitter: @RSocPublishing
Follow Royal Society Publishing on Facebook:
https://www.facebook.com/RoyalSocietyPublishing.FanPage/
Read Royal Society Publishing's blog:
https://royalsociety.org/blog/blogsearchpage/?category=Publishing
